# Region of Employment and Intention to Remain Practicing or Exit the Profession Among Australian Nurses: A Cross‐Sectional Analysis

**DOI:** 10.1155/jonm/4278991

**Published:** 2026-01-29

**Authors:** Maureen Dillon, Jane Mills, Lisa Hanson, Helen Wright, George Mnatzaganian

**Affiliations:** ^1^ Department Rural Health Sciences, La Trobe Rural Health School, La Trobe University, Bendigo, Victoria, Australia, latrobe.edu.au; ^2^ La Trobe Rural Health School, La Trobe University, Bendigo, Victoria, Australia, latrobe.edu.au; ^3^ Department Rural Allied Health, La Trobe Rural Health School, La Trobe University, Bendigo, Victoria, Australia, latrobe.edu.au; ^4^ College of Healthcare Sciences, James Cook University, Townsville, Queensland, Australia, health.qld.gov.au

**Keywords:** intention to leave, intention to stay, nurse education, nursing workforce, region, resilience, rurality

## Abstract

**Aim:**

This cross‐sectional study explored factors influencing nurses’ intention to remain practicing or exit the profession across Australian metropolitan, regional, rural, and remote settings to inform workplace policies, education training, and career coaching.

**Background:**

Evidence on attrition and retention across geographical regions of employment is mixed. While much existing research on the nursing workforce has focused on job satisfaction, work–life balance, and workload, less attention has been given to the influence of work setting, nursing role, resilience, and career advancement opportunities—particularly in nonmetropolitan areas.

**Methods:**

Validated scales were used to assess resilience and intention to stay in the profession. Intention to stay was analyzed using ordinal logistic regression and Youden’s statistic was applied to estimate the year nurses were most likely to consider leaving.

**Results:**

Of 1252 survey accesses, 526 (42.0%) resulted in completion and 410 (77.9%) included a response to the intention‐to‐stay question. No statistically significant differences were found in age, gender, early career status, or years of experience among nurses working in different geographic areas. Using Youden’s statistical method, 7 years of employment was identified as the threshold at which nurses were most likely to consider leaving their profession. Compared with metropolitan nurses, those in rural/remote areas were more likely to stay (adjusted odds ratio: 2.2; 95% CI: 1.14–4.10; and *p* = 0.019). Positive predictors included working in clinical roles, hospital or community settings, higher resilience, and career advancement opportunities.

**Conclusions:**

Region, role, and resilience strongly influence nurses’ intention to stay, with rural and remote nurses more likely to remain than metropolitan peers.

**Implications for Nursing Management:**

Targeted supports—including mentorship, reflective practice, resilience training, and career coaching—may improve retention, particularly when management practices are tailored to leverage the strengths of rural and remote settings while addressing context‐specific needs to enhance workforce stability across all regions.

## 1. Introduction

Nurses play a vital role in delivering healthcare services across Australia, yet ensuring workforce stability and sustainability remains a significant and growing challenge. Although the number of nurses and midwives in Australia increased by 30% between 2015 and 2023 [1], demand continues to exceed supply. A projected shortfall of 70,707 full‐time equivalent (FTE) positions—equivalent to 79,473 nurses—is expected by 2035 [2]. These shortages are likely to disproportionately impact regional, rural, and remote areas, where access to healthcare is already limited [3]. Meeting workforce demand remains a challenge due to factors such as an aging workforce, high turnover, and limited entry of new nurses into the profession. In Australia, turnover rates vary widely [4–6], reaching up to 148% in remote areas and placing operational and financial strain on healthcare services [7], especially in nonmetropolitan regions [8]. Beyond the operational disruptions, high turnover affects continuity of care and increases pressure on remaining staff.

Resilience has emerged as a critical factor in nurse retention. While job satisfaction, work–life balance, and organizational support are important, the ability to cope with workplace stress, recover from burnout, and adapt to ongoing demands significantly influences nurses’ decisions to stay in their roles [9–11]. Studies consistently highlight that enhancing resilience not only supports nurse well‐being but also strengthens professional commitment and longevity [12]. Addressing burnout and fostering supportive, resilient workplaces are therefore essential to stabilizing the nursing workforce [13–15].

Workforce challenges in regional, rural, and remote areas of Australia are further complicated by geographic isolation, limited access to professional development, and reduced social and professional support networks [16]. Nurses working in these settings often face heavier workloads and fewer career advancement opportunities [17, 18], while health services face persistent difficulties in attracting and retaining qualified staff [19]. These factors, coupled with inadequate support structures and constrained career pathways, contribute to elevated turnover rates in nonmetropolitan regions.

While there is substantial empirical evidence suggesting that nurses and midwives in rural and regional areas are at greater risk of leaving their position or location than their metropolitan counterparts—particularly in small, remote, or poorly resourced settings [5, 8], or among younger or full‐time staff—the overall evidence is mixed and highly context‐dependent. Factors such as country, service type, the presence of a local hospital, and employment status (full‐time versus part‐time) all influence retention outcomes. Some studies indicate that rural retention is achievable under supportive conditions; for example, Sellers et al. [20] reported that job satisfaction, opportunities for professional growth, and alignment with rural lifestyle can mitigate known challenges. Similarly, a recent large U.S. study involving 14,986 nurses found higher registered nurse (RN) turnover in urban skilled‐nursing facilities (51.5%) compared with rural facilities (47%) [21]. As Australia’s population ages and demand for healthcare rises—particularly in underserved rural areas—gaining insight into the factors that influence nurse retention is critical to ensuring a resilient and sustainable healthcare workforce. Despite the growing urgency, there remains a lack of comprehensive research specifically exploring the factors that support retention of nurses employed in regional and rural settings. This cross‐sectional study addresses that gap by investigating the determinants of nurses’ intentions to continue practicing or to exit the profession, with a focus on geographic variation across metropolitan, regional, rural, and remote locations.

## 2. Methods

### 2.1. Ethics Statement

The study was approved by the La Trobe University Human Research Ethics Committee (HEC 22048). The online questionnaire included an information and consent statement, which also asked participants for optional permission to be contacted for a follow‐up interview. Completion of the survey indicated consent to participate.

### 2.2. Study Design, Scales, and Population

This cross‐sectional survey was part of a broader multiple case study that included both an online questionnaire and follow‐up semistructured interviews with participants who provided consent. The multiple case study design examined three geographic regions—metropolitan, regional, and rural—to provide a deeper, comparative understanding of nurses’ intention to stay in the profession. By analyzing each case individually and then comparing patterns across cases, the design helped explain how and why influences on retention differed or aligned across settings. The anonymous questionnaire consisted of four sections: demographics and three validated tools—the Connor–Davidson Resilience Scale [22], the Practice Environment Scale of the Nursing Work Index [23], and the Nurse Retention Index [24]. The survey, hosted on the QuestionPro platform, was promoted via social media channels such as Facebook and LinkedIn, as well as at a conference hosted by the Australian College of Nursing in May 2023. The survey was administered to enrolled nurses, registered nurses, midwives, and nurse practitioners holding current registration with Australian Health Practitioner Regulation Agency (AHPRA).

The findings presented in this manuscript are based solely on the results from the quantitative questionnaire survey.

### 2.3. Sample Size Estimation

**Figure 1:**
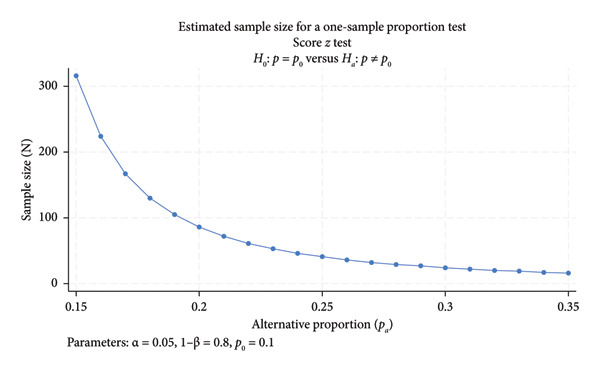
Sample size needed by different exit and intention to leave proportions.

The Australian Department of Health, Disability, and Aging Nursing Supply and Demand Study reports a national exit rate from the nursing profession of approximately 10% [25]. In rural regions, the exit rate is estimated to be around 20% [26], with intention to leave the profession reported at approximately 34% [27]. Using the national exit rate of 10% and assuming a lower than above mentioned rural exit rates of 15% and applying a two‐sided *α* of 0.05 and power of 0.80, our study required a maximum sample size of 315 nurses (Figure 1).

### 2.4. Statistical Methods

Participants were asked to complete a survey regarding their satisfaction with the current nursing practice environment, the support available, opportunities for career advancement, perceived leadership at the workplace, patient care and clinical competence, interpersonal relationships at the workplace, their resilience, and their intention to stay in or leave the nursing workforce. Study variables included age, gender, years of clinical experience, residential postcode, postcode of primary place of employment, principal role, primary work location (hospital based, nonhospital based, and other organization), and main specialty area of practice, as well as questions derived from the study scales.

Using the Nurse Retention Index [24], participants were asked to provide the most appropriate response, ranging from “definitely false” to “definitely true.” The intention to stay questions related to the following statements:

• “It is my intention to continue with my nursing career in the foreseeable future.”
• “I would like to stay in nursing as long as possible.”
• “I expect I will keep working as a nurse.”
• “My plan is to remain with my nursing career as long as I am able.”

The intention to leave related to the following statements:

• “As soon as it is convenient for me, I plan to leave the nursing profession.”
• “I would like to find other employment by leaving nursing.”

Median values were used to summarize responses regarding nurses’ intention to remain in or exit the profession. Differences in nurse characteristics by employment location were assessed using chi‐squared and Kruskal–Wallis tests for categorical and continuous variables, respectively. Participants’ intention to continue in the nursing career was plotted by employment region.

Youden’s J statistic [28] was used to determine the optimal threshold year after which a nurse was likely to consider leaving the profession. To accomplish this, the overall median score of the questions assessing the intention to leave was dichotomized into two categories: responses including “definitely false,” “false,” “mostly false,” and “more false than true” were categorized as “no,” while responses including “more true than false,” “mostly true,” “true,” and “definitely true” were categorized as “yes.” A sensitivity analysis was conducted that categorized the responses of “true” and “definitely true” against other responses.

The overall median score for the intention to continue working in the nursing profession ranged from 1 to 8, with 1 indicating “definitely false” and 8 indicating “definitely true.” This was further analyzed using ordinal logistic regression, which compared employment regions while adjusting for gender, years of experience, social status (measured by the Relative Socioeconomic Disadvantage (IRSD) [29]), clinician status, work setting, principal job area, career advancement opportunities at the workplace, and the nurse’s personal attribute of resilience. Questions related to patient care, clinical competence, perceived leadership at the workplace, and interpersonal relationships showed strong correlations (Spearman’s rho ≥ 0.4) with resilience and career advancement questions and were, therefore, excluded from the multivariable model. The model demonstrating the best fit was selected using the lowest Akaike’s Information Criterion (AIC) [30].

Artificial intelligence (AI) was not used in the Methods or any other section of this manuscript.

## 3. Results

**Figure 2:**
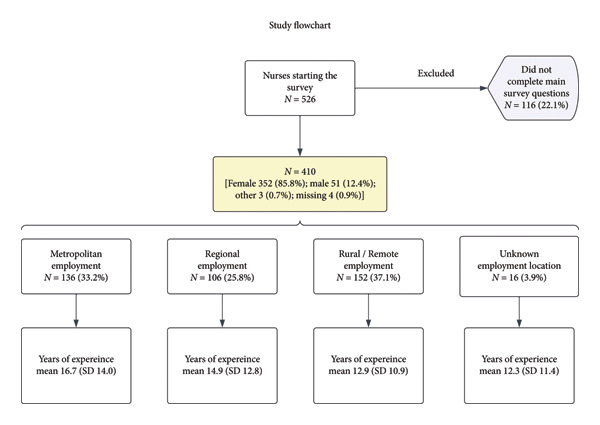
Study flowchart.

The survey was viewed by 1252 individuals, with 526 participants, resulting in a response rate of 42.0%. Of the 526 nurses who started the survey, 410 (77.9%) answered questions related to their intention to remain practicing or exit the nursing profession (Figure 2). The 116 participants who were excluded for not answering the main study questions were significantly younger and had a higher proportion of males compared with those who completed the questions (*p* < 0.05 for both comparisons). No statistical differences were observed between the groups in terms of years of experience or region of employment.

No statistical differences were observed in age, gender, early career status, years of experience, or primary work setting among nurses working in metropolitan, regional, rural, or remote areas (Table 1). However, compared with those in metropolitan areas, nurses working in regional, rural, or remote locations were more likely to come from lower socioeconomic backgrounds (*p* < 0.001) and to work in aged care or community nursing (*p* = 0.001). Clinical work was most observed among nurses in regional areas (65.1%), compared with 52.2% in metropolitan areas and 56.6% in rural or remote areas (*p* < 0.001).

**TABLE 1:** Characteristics of participants by region of employment.

	All	Metropolitan	Regional	Rural/remote	**p**​ value^∗^
*n* (%)	394 (100%)	136 (34.5%)	106 (26.9%)	152 (38.6%)	
Age, mean (SD), median [IQR]	43.7 (12.5), 43 [33, 55]	43.7 (13.2), 42 [32, 55]	43.6 (11.6), 43 [34, 53.5]	44.5 (12.6), 45 [33, 56]	0.752
Gender, *n* (%)					0.553
Female	343 (87.1)	117 (86.0)	91 (85.8)	135 (88.8)	
Male	45 (11.4)	18 (13.2)	13 (12.3)	14 (9.2)	
Other	3 (0.8)	0 (0.0)	2 (1.9)	1 (0.7)	
Unknown	3 (0.8)	1 (0.7)	0 (0.0)	2 (1.3)	
SEIFA‐IRSD, n (%)					< 0.001
1st quintile (most disadvantaged)	74 (18.8)	12 (8.8)	15 (14.1)	47 (30.9)	
2nd quintile	106 (26.9)	16 (11.8)	47 (44.3)	43 (28.3)	
3rd quintile	83 (21.1)	19 (14.0)	23 (21.7)	41 (27.0)	
4th quintile	60 (15.2)	31 (22.8)	14 (13.2)	15 (9.9)	
5th quintile (least disadvantaged)	60 (15.2)	55 (40.4)	2 (1.9)	3 (2.0)	
Unknown	11 (2.8)	3 (2.2)	5 (4.7)	3 (2.0)	
Early career, *n* (%)					0.805
Yes	125 (31.7)	43 (31.6)	31 (29.2)	51 (33.6)	
No	261 (66.2)	92 (67.6)	71 (67.0)	98 (64.5)	
Unknown	8 (2.0)	1 (0.7)	4 (3.8)	3 (2.0)	
Years of experience, mean (SD), median [IQR]	14.7 (12.6), 11 [4, 23]	16.7 (14.0), 14 [4, 29]	14.9 (12.8), 10 [5, 23]	12.9 (11.0), 10 [5, 20]	0.358
Principal role, *n* (%)					< 0.001
Clinician	226 (57.4)	71 (52.2)	69 (65.1)	86 (56.6)	
Administrator	33 (8.4)	14 (10.3)	3 (2.8)	16 (10.5)	
Educator	40 (10.1)	13 (9.6)	16 (15.1)	11 (7.2)	
Researcher	13 (3.3)	9 (6.6)	2 (1.9)	2 (1.3)	
Other	39 (9.9)	24 (17.6)	6 (5.7)	9 (5.9)	
Unknown	43 (10.9)	5 (3.7)	10 (9.4)	28 (18.4)	
Primary work setting, *n* (%)					0.336
Hospital based	248 (62.9)	87 (64.0)	71 (67.0)	90 (59.2)	
Nonhospital based	58 (14.7)	20 (14.7)	17 (16.0)	21 (13.8)	
External organizations	10 (2.5)	6 (4.4)	2 (1.9)	2 (1.3)	
Education institutions	14 (3.6)	6 (4.4)	3 (2.8)	5 (3.3)	
Other	19 (4.8)	12 (8.8)	2 (1.9)	5 (3.3)	
Unknown	45 (11.4)	5 (3.7)	11 (10.4)	29 (19.1)	
Main area of work, *n* (%)					0.001
Mental health	48 (12.2)	18 (13.2)	21 (19.8)	9 (5.9)	
Emergency/ICU	43 (10.9)	19 (14.0)	8 (7.5)	16 (10.5)	
Maternity care	40 (10.1)	13 (9.6)	12 (11.3)	15 (9.9)	
Aged care	24 (6.1)	4 (2.9)	11 (10.4)	9 (5.9)	
Medical/surgical	20 (5.1)	7 (5.1)	1 (0.9)	12 (7.9)	
Perioperative	15 (3.8)	5 (3.7)	4 (3.8)	6 (3.9)	
Community nursing	24 (6.1)	4 (2.9)	7 (6.6)	13 (8.5)	
Education	18 (4.6)	4 (2.9)	8 (7.5)	6 (3.9)	
Management/policy	29 (7.4)	16 (11.8)	2 (1.9)	11 (7.2)	
Other	90 (22.8)	41 (30.1)	22 (20.7)	27 (17.8)	
Unknown	43 (10.9)	5 (3.7)	10 (9.4)	28 (18.4)	

*Note:* Socioeconomic Indices for Areas—Index of Relative Socioeconomic Disadvantage (SEIFA‐IRSD).

^∗^The “unknown” was not included in the statistical comparisons of the groups.

**Figure 3:**
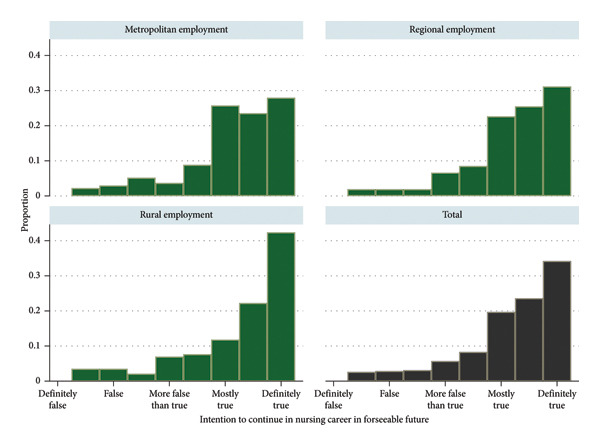
Intention to remain practicing as a nurse in the foreseeable future by region of employment.

The majority of nurses (*n* = 350; 85.4%) reported an intention to remain in the profession, while 62 nurses (15.1%) indicated that they were considering leaving in the foreseeable future. Among these 62 nurses, years of experience ranged from 1 to 45 years, with between 25.0% and 45.5% having no more than 7 years of experience. Intention to stay working as a nurse in the foreseeable future varied by region, with nurses employed in rural and remote areas showing a more positive outlook. However, the unadjusted group comparisons did not reach statistical significance (*p* = 0.057) (Figure 3).

The analysis using Youden’s index indicated that after 7 years of employment, nurses were more likely to consider leaving the profession. The sensitivity analysis, which focused only on the “true” and “definitely true” responses, produced similar results.

**Figure 4:**
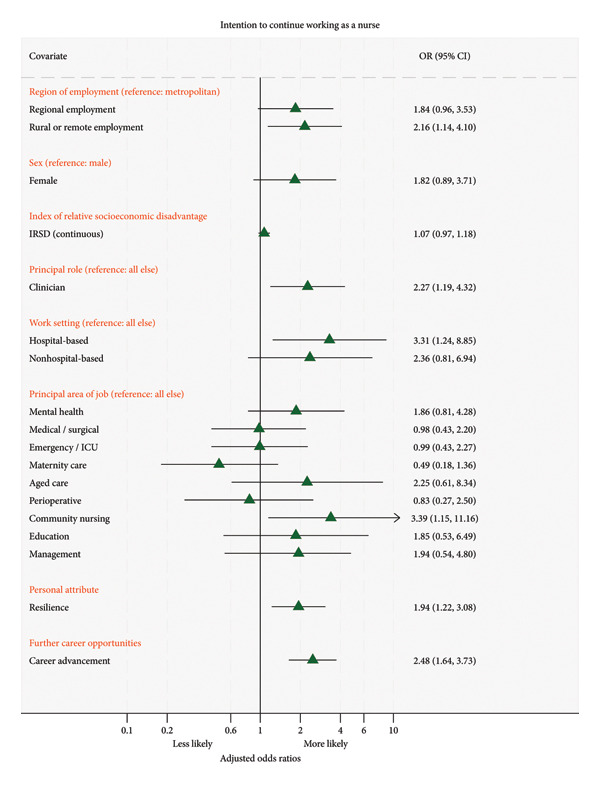
Factors associated with the intention to continue practicing as a nurse: multivariable regression analysis.

In the multivariable analysis that adjusted for the covariates shown in Figure 4, nurses working in rural or remote locations were 2.16 times more likely to continue working in the profession compared to their counterparts in metropolitan regions (adjusted odds ratio [AOR]: 2.2, 95% confidence interval [CI]: 1.1–4.1, and *p* = 0.019). Other factors associated with the intention to continue working as a nurse included being a clinician (AOR: 2.3, 95% CI: 1.2–4.3, and *p* = 0.012), working in a hospital (AOR: 3.3, 95% CI: 1.2–8.8, and *p* = 0.017), working in community nursing (AOR: 3.6, 95% CI: 1.2–11.2, and *p* = 0.028), having resilience (AOR: 1.9, 95% CI: 1.2–3.1, and *p* = 0.005), and having opportunities for career advancement (AOR: 2.5, 95% CI: 1.6–3.7, and *p* < 0.001).

## 4. Discussion

This cross‐sectional study explored the factors that influence nurses’ decision to leave or stay in the workforce focusing on metropolitan, regional, rural, and remote employment locations. No statistical differences were found in age, gender, early career status, years of experience, or primary work setting among nurses from different regions. The analysis indicated that after 7 years of employment, nurses were more likely to consider leaving the profession. In the multivariable analysis, nurses in rural or remote areas were more likely to stay in the profession, with other factors such as being a clinician, working in a hospital or community nursing, resilience, and opportunities for career advancement also positively influencing their intention to remain in the workforce.

In regional and rural health settings—defined here as areas located beyond metropolitan centers—nurses play a pivotal role in providing healthcare to the community. This is particularly true in Australia’s remote and isolated regions, where nurses represent the largest segment of the healthcare workforce [31]. Their responsibilities extend across the lifespan, delivering essential care and tailored health services to meet the diverse needs of local populations [31, 32]. However, the nursing workforce shortage in Australia [2], and especially in rural Australia [33], poses a critical threat to these communities. Declining enrollment in nursing education, coupled with increased healthcare demand from an aging population, is exacerbating workforce pressures [34]. Rural and remote areas are especially vulnerable, given the persistent challenges they face, including unequal distribution of health professionals, ongoing staffing shortages, and elevated turnover rates [8, 21]. These workforce dynamics contribute significantly to the broader health inequities experienced in these regions. Contrary to previous research [19, 35], our study found that nurses in rural and regional areas reported greater intention to remain in the nursing profession than their metropolitan counterparts. This suggests that despite resource limitations and service delivery challenges often associated with rural practice [36], rural nurses may experience stronger commitment to their roles and communities. Factors such as close‐knit community relationships, professional autonomy, and broader scope of practice likely contribute to these positive retention intentions. This finding also suggests that rural nurses may derive a greater sense of purpose, professional fulfillment, and identity alignment in their roles, serving as essential contributors to their communities and often delivering a broad spectrum of care across the lifespan in settings where their presence is vital [37, 38]. In contrast, metropolitan nurses may experience greater workforce churn due to a combination of increased employment competition, fragmented workplace environments, and more accessible alternative career opportunities [39]. The greater density of healthcare facilities in urban areas contributes to this competition and when coupled with high workloads and unsupportive work environments can lead nurses to frequently transition between positions in search of better pay, improved conditions, or career advancement [40]. A study focusing on cancer nurses further highlights that intense job strain—driven by heavy workloads and the demand for high levels of psychosocial care—can significantly increase turnover intentions more commonly seen in metropolitan regions [41].

Recent data reveal a concerning pattern in nurse retention, with a significant proportion of nurses intending to leave their current roles or the profession entirely. According to the Australian Primary Health Care Nurses Association [1], one in four nurses considers leaving their positions within 2–5 years, and nearly one in ten is contemplating departure within the next year. Early career nurses—those with one to 4 years of experience—appear particularly vulnerable, with approximately one‐third of registered nurses exiting the workforce within their first 2 years of practice [42]. Our study identified a critical 7‐year threshold, beyond which nurses become more likely to consider leaving the profession. This point may coincide with career reflection [43], shifting personal circumstances, increased caring responsibilities, or the onset of burnout. These patterns point to a critical juncture in the nursing career trajectory where targeted interventions could make a meaningful difference in workforce stability. Addressing the underlying drivers of attrition—such as dissatisfaction, disengagement, and burnout—through structured support, opportunities for professional advancement, leadership development, and adaptable work arrangements may help sustain nurses’ engagement and strengthen their long‐term commitment to the profession. Implementing tailored strategies at this stage could play a key role in improving retention and reinforcing career satisfaction.

Our study found that career retention among nurses is influenced by both the nature of their work and the setting in which they are employed. Clinician nurses were more likely to express an intention to remain in the profession compared to their nonclinician counterparts, with this intention particularly strong among those working in hospital (acute care) settings. Conversely, we also report that nurses in nonacute or community‐based roles may have longer actual tenure. This highlights a complex dynamic in retention patterns—suggesting that while acute care environments may foster stronger professional engagement and perceived commitment, nonacute roles may offer greater longevity, perhaps due to different work demands or career trajectories [44, 45]. Given Australia’s aging population and the growing demand for out‐of‐hospital care, urgent policy attention is required to enhance workforce sustainability in community‐based settings. Targeted strategies to support and retain nurses in these sectors are essential to securing a stable and resilient nursing workforce for the future.

Our study also identified resilience as a key factor influencing nurses’ intention to remain in the profession. Nurses with higher resilience are more likely to cope effectively with workplace demands, reduce burnout, and maintain long‐term commitment to their roles [14, 46]. Supporting resilience through targeted interventions may help improve retention across diverse healthcare settings. While resilience is often framed as a desirable attribute in nursing, recent scholarship challenges the assumption that resilience is universally beneficial. Suslovic and Lett [47] argue that resilience can function as an “adverse event” when it shifts responsibility for coping onto individuals rather than addressing the structural and organizational conditions that generate stress and burnout. Within nursing, this critique is particularly relevant: emphasizing individual resilience may inadvertently normalize excessive workloads, chronic understaffing, and exposure to occupational trauma, implying that nurses should adapt to harmful conditions rather than expecting those conditions to improve. At the same time, our findings indicate that resilience remains an important personal resource, with more resilient nurses demonstrating a greater intention to remain in the profession. This suggests that resilience may support workforce stability, but it should not be viewed as a substitute for organizational reform. Instead, resilience‐building efforts should occur alongside systemic strategies that create safer, more sustainable working environments.

This research has direct implications for nurse education, professional development, and workforce planning. Understanding the timing—specifically the 7‐year threshold—and underlying factors that contribute to nurses’ intention to leave the profession enables educators and employers to intervene proactively. Educational curricula and continuing professional development (CPD) programs can be designed to better prepare nurses for the challenges commonly encountered in different stages of their careers, particularly beyond the early years of practice when vulnerability to attrition appears to increase [15].

Targeted support mechanisms, such as structured mentorship, reflective practice, resilience training, and career coaching, could be integrated into both preregistration education and ongoing in‐service programs. These initiatives may help nurses develop coping strategies, strengthen professional identity, and foster a sense of long‐term career progression [37]. For example, nurse educators and clinical leaders might collaborate to embed discussions of career planning and self‐care into professional development pathways, particularly for those approaching midcareer stages.

Furthermore, the identification of contextual factors that promote retention—such as opportunities for career advancement, meaningful clinical roles, and rural employment settings—highlights the importance of tailoring retention strategies to specific employment environments [11]. Workforce policy could be informed by these findings to allocate resources strategically, enhance job satisfaction, and provide clear advancement opportunities, especially in underserved rural and remote areas where retention is often a greater challenge [20, 21]. Ultimately, aligning educational strategies with workforce needs can contribute to a more sustainable nursing workforce by reducing attrition and improving long‐term career satisfaction across the profession.

While our study highlights several strategies that may support retention in the nursing workforce, their successful implementation depends on broader organizational and system‐level enablers. Improving retention in rural settings requires investment in training for nurse managers and rural healthcare administrators [48], strengthened resources for rural health services [49], and formal recognition of staff development within organizational performance frameworks [50]. Embedding workforce development indicators into routine reporting may help ensure accountability and sustained attention to retention initiatives. These structural supports are essential to complement individual‐level strategies and create conditions in which nurses are more likely to remain in the profession.

## 5. Limitations

This study has several limitations inherent to cross‐sectional survey designs. The response rate was relatively low at 42%, raising concerns about potential nonresponse bias. Additionally, the sample may not be fully representative of the broader nursing workforce, particularly regarding the distribution of nurses across different roles and settings. Some groups, such as nurses in certain clinical or nonclinical roles, may have been under‐ or overrepresented, which could affect the generalizability of the findings. While the study participants were recruited via social media and a conference, we were unable to determine how many participants were recruited via each platform because the online survey did not collect information on the recruitment method. Finally, Youden’s Index does not incorporate the underlying prevalence of the outcome being assessed, and by design, it yields only a single optimal cut‐point [28]. In contexts where the phenomenon may have multiple meaningful thresholds or differ across population subgroups, this can oversimplify interpretation.

## 6. Conclusion

Our findings challenge the assumption that rural and regional nurses are more likely to leave the profession. On the contrary, their stronger intention to stay points to the potential of these nurses as a stable, committed workforce segment. To capitalize on this potential, targeted investments are needed to further support rural nurses, including improved access to education, flexible career pathways, and initiatives that promote well‐being and resilience. This study highlights the complexity of factors influencing nurses’ intention to stay in the profession. Notably, we found that nurses in rural and regional areas were more likely than metropolitan nurses to stay in their nursing career, suggesting that contextual and personal factors may outweigh structural challenges in these regions. Work setting, role type, and opportunities for development also emerged as important influences.

## 7. Implications for Nursing Management

These findings emphasize the need for regionally tailored workforce strategies that recognize local strengths while addressing sector‐specific challenges. Sustaining a motivated and committed nursing workforce across all settings is essential for meeting Australia’s current and future healthcare needs. In regional and rural settings, where nurses often face unique challenges such as professional isolation, heavier workloads, and limited resources, management must tailor approaches to leverage the inherent strengths of these communities—such as strong team cohesion and commitment to local care. Developing locally relevant support structures and flexible career development opportunities can address specific barriers faced by nurses in these areas. By doing so, nursing leadership can enhance workforce stability, improve retention rates, and ensure continuity of care across all geographic regions.

## Funding

No funding was received for this manuscript.

## Conflicts of Interest

The authors declare no conflicts of interest.

## Data Availability

The data that support the findings of this study are available from the corresponding author upon reasonable request.
